# Predictive Factors for Subjective Improvement in Lumbar Spinal Stenosis Patients with Nonsurgical Treatment: A 3-Year Prospective Cohort Study

**DOI:** 10.1371/journal.pone.0148584

**Published:** 2016-02-10

**Authors:** Ko Matsudaira, Nobuhiro Hara, Hiroyuki Oka, Junichi Kunogi, Takashi Yamazaki, Katsushi Takeshita, Seichi Atsushi, Sakae Tanaka

**Affiliations:** 1 Department of Medical Research and Management for Musculoskeletal Pain, 22nd Century Medical & Research Center, Faculty of Medicine, University of Tokyo, Tokyo, Japan; 2 Department of Orthopaedic Surgery, Musashino Red Cross Hospital, Tokyo, Japan; 3 Department of Spine and Orthopaedic Surgery, Japanese Red Cross Medical Center, Tokyo, Japan; 4 Department of Orthopaedic Surgery, Jichi Medical University, Tochigi, Japan; 5 Department of Orthopaedic Surgery, Mitsui Memorial Hospital, Tokyo, Japan; 6 Department of Orthopaedic Surgery, University of Tokyo, Tokyo, Japan; University of Texas Health Science Center at Houston, UNITED STATES

## Abstract

**Objective:**

To assess the predictive factors for subjective improvement with nonsurgical treatment in consecutive patients with lumbar spinal stenosis (LSS).

**Materials and Methods:**

Patients with LSS were enrolled from 17 medical centres in Japan. We followed up 274 patients (151 men; mean age, 71 ± 7.4 years) for 3 years. A multivariable logistic regression model was used to assess the predictive factors for subjective symptom improvement with nonsurgical treatment.

**Results:**

In 30% of patients, conservative treatment led to a subjective improvement in the symptoms; in 70% of patients, the symptoms remained unchanged, worsened, or required surgical treatment. The multivariable analysis of predictive factors for subjective improvement with nonsurgical treatment showed that the absence of cauda equina symptoms (only radicular symptoms) had an odds ratio (OR) of 3.31 (95% confidence interval [CI]: 1.50–7.31); absence of degenerative spondylolisthesis/scoliosis had an OR of 2.53 (95% CI: 1.13–5.65); <1-year duration of illness had an OR of 3.81 (95% CI: 1.46–9.98); and hypertension had an OR of 2.09 (95% CI: 0.92–4.78).

**Conclusions:**

The predictive factors for subjective symptom improvement with nonsurgical treatment in LSS patients were the presence of only radicular symptoms, absence of degenerative spondylolisthesis/scoliosis, and an illness duration of <1 year.

## Introduction

Lumbar spinal stenosis (LSS) presents with neurological symptoms, such as numbness, pain, and intermittent claudication, in the lower extremities due to a narrowing of the intervertebral foramen and spinal canal, which serve as a passageway for nerves in the lumbar region.[1] Because of these symptoms, LSS is an important risk factor for decreased quality of life (QOL), particularly in the elderly. Previous epidemiological studies in Japan indicated a prevalence of LSS among people aged ≥70 years of approximately 10%.[2] With the aging society, the number of patients with LSS is predicted to rapidly increase. Thus, LSS is a disease that will be frequently encountered by primary care physicians.

With regard to LSS prognosis, several reports have demonstrated better outcomes with surgery compared with nonsurgical treatments.[3–5] Conversely, various other reports have revealed that, in some patient groups with relatively mild symptoms, the disease’s natural course has a favourable prognosis.[6–10] However, patients with mild symptoms were excluded from some studies, and, in other cases, patients with severe symptoms requiring surgery were excluded. Therefore, it is not possible to draw conclusions regarding the natural history of LSS in all patients. To determine which patients have favourable prognoses, studies need to be conducted on a wide range of patients with LSS, regardless of the disease severity and therapeutic methods. However, to the best of our knowledge, no such study has been conducted.

Our hypothesis was that pre-treatment factors, such as duration of illness, types of symptoms, radiographic features, comorbidity, would predict patients’ subjective improvement without surgical intervention. The aim of this study was to establish the evidence for favourable prognoses without surgical intervention.

## Materials and Methods

### Study design

This study was an investigator-initiated observational cohort study conducted at 17 medical centres in Japan, in which a wide variety of treatments, including surgical and conservative methods, were used in the treatment of spinal diseases. This study was approved by institutional review board of University of Tokyo, Tokyo Metropolitan Geriatric Hospital, Hitachi General Hospital, Asama General Hospital, MIshuku Hospital, Musashino Red Cross Hospital, Tokyo Metropolitan Tama Synthesis Medical Center, Japanese Red Cross Medical Center, Tokyo Yamate Medical Center, NTT Medical Center Tokyo, Sanraku Hospital, Kanto Central Hospital, Tokyo Metropolitan Hiroo Hospital, Tokyo Metropolitan Komagome Hospital, Kosei Hospital, Yokohama Rosai Hospital, Toranomon Hospital, and written informed consent was obtained from all participants.

### Patient population

Patients with LSS were enrolled from the University of Tokyo Hospital and 17 related facilities between July 2002 and June 2003 based on the following eligibility criteria: aged 50–85 years old and LSS based on the definition of Verbiest [11] (presence of paraesthesia or pain in the lower extremities, buttocks, perineum, or perianal region and magnetic resonance imaging showing the presence of spinal canal stenosis that may explain the patient’s symptoms). Based on the pathogenesis, the patient’s condition was required to be degenerative acquired stenosis (e.g., spondylosis, spondylolisthesis, or scoliosis), and patients with congenital, developmental, or post-traumatic LSS as well as those who underwent spinal surgery were excluded. The exclusion criteria were also as follows: presence of lumbar disc herniation (i.e., a positive straight leg raise test); arteriosclerosis obliterans (i.e., non-palpable foot arteries); complications causing disorders that interfere with gait, such as those after cerebral infarction or myelopathy; diagnosis of lower extremity symptoms because of peripheral nerve diseases; rheumatoid arthritis or Parkinson's disease; current administration of psychosomatic medicine or outpatient treatment at a psychiatric department; and compensation for damage.

Of the 314 patients that were screened, the study enrolled 274 patients (151 men, 123 women; mean age, 71 years) whose eligibility was guaranteed by a third-party evaluation.

In this study, a database was created by prospectively enrolling patients with LSS, regardless of the disease severity or treatment. Three years later, their prognosis was examined, and the factors that led to a subjective improvement in their symptoms without surgical intervention were assessed.

### Study interventions

The treatment choice was made by the patients and physicians of each facility. The therapeutic methods included surgery (i.e., posterior lumbar decompression, posterior lumbar spinal fusion, or anterior lumbar interbody fusion) and nonsurgical methods (i.e., administration of non-steroidal anti-inflammatory drugs or prostaglandin E1 derivatives, exercise therapy, physical therapy, or nerve blocks). There was no limitation to the treatment selection.

### Study measures

The following variables were examined at initial enrolment: degree of obesity (body mass index: ≥25 or <25 kg/m^2^), educational background (at least a high school graduate, other), current comorbidities (hypertension, diabetes mellitus), duration of illness (<12 months, 12–59 months, or ≥60 months), types of symptoms (presence of cauda equina symptoms, at least the presence of bilateral numbness in the lower limbs), and presence of degenerative spondylolisthesis (% slip ≥5%) and scoliosis (Cobb angle ≥10 degrees) on radiographs. In addition, the Geriatric Depression Scale (GDS)-15, which is the abridged version of the GDS-30, was administered and assigned to tertiles defined by approximate thirds of the score distribution (0–2, 3–6, and ≥7) to assess depression.[12]

Three years after enrolment, a self-administered survey was delivered by mail to examine the patients’ subjective improvement and determine whether surgery had been performed. In addition, the study centre also contacted survey non-respondents by telephone as an alternative form of contact to increase the response rates. The subjective degree of improvement was based on a 5-point scale, with 1 and 2 points indicating improvement without surgical intervention: 1) the condition has improved a lot; 2) the condition has improved; 3) nothing has changed; 4) the condition has become worse; and 5) the condition has become a lot worse.

### Statistical analysis

A multivariable logistic regression model was used to assess the relationship between the candidate variables and patients’ subjective improvement without surgical intervention. The following candidate variables were included in the final regression model when P < 0.10 in the univariable analysis: age, sex, obesity, educational background, duration of illness, types of symptoms, and the presence of each of degenerative spondylolisthesis/degenerative scoliosis, hypertension, diabetes, and depression (GDS-15). Statistical analyses were performed using SPSS version 20.0 (IBM Corp., Armonk, NY, USA). A P value < 0.05 was considered to be statistically significant, and all reported P values are two sided.

## Results

The 3-year follow-up rate was 67.5% (n = 185). There were no differences in the candidate variables between the 185 patients who completed the follow-up survey and the 89 patients who did not (Table 1).

**Table 1:** Baseline characteristics, compared between the participants with lumbar spinal stenosis who did and did not complete the 3-year follow-up.

	Participants	Drop-outs	P-value
	(n = 185)	(n = 89)	
Age (years), mean (SD)	70.7 (7.4)	71.7 (7.6)	0.28
BMI (kg/m^2^), mean (SD)	23.4 (3.1)	23.2 (3.1)	0.53
Gender (%)			
Female	77 (41.6)	46 (51.7)	0.12
Educational background			
At least a high school graduate	134 (72.4)	64 (71.9)	0.93
Cauda equina symptoms	78 (42.2)	44 (49.4)	0.26
Degenerative spondylolisthesis/scoliosis	99 (53.5)	47 (47.5)	0.91
Duration of illness (months)			
<12	48 (26.0)	23 (25.8)	0.99
12–59	80 (43.2)	38 (42.7)	
≥60	57 (30.8)	28 (31.5)	
Hypertension	120 (64.9)	57 (64.0)	0.89
GDS score (tertiles)			
0–2	73 (39.5)	27 (30.3)	0.13
3–6	64 (34.6)	29 (32.6)	
≥7	48 (25.9)	33 (37.1)	

BMI, body mass index; SD, standard deviation; GDS, Geriatric Depression Scale

The values are reported as n (%), unless indicated.

**Fig 1:**
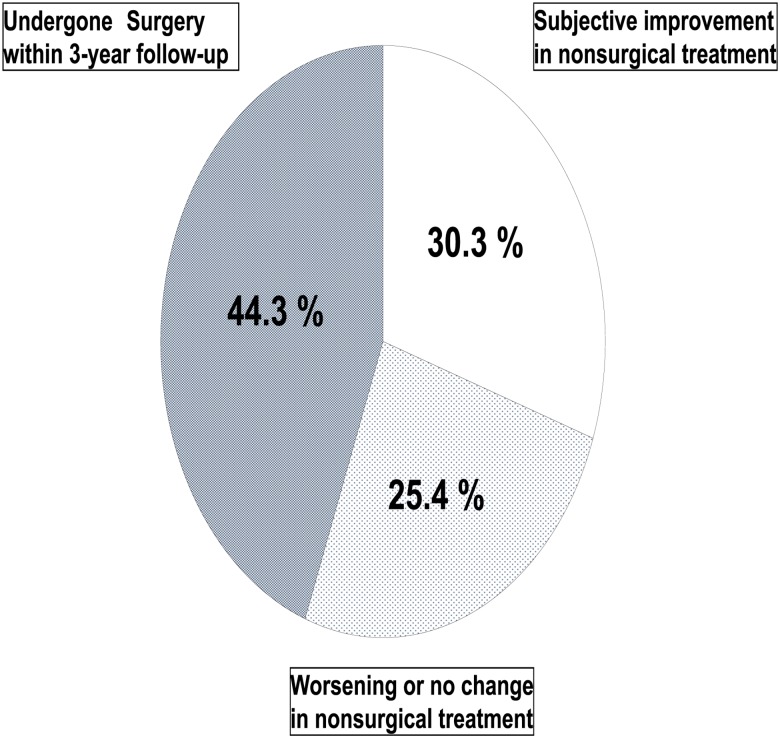
Response to the self-administered survey in 185 patients with lumbar spinal stenosis 3 years after treatment.

Nonsurgical treatment resulted in subjective improvements in 56 (30.3%) of the 185 patients, and the condition worsened or did not change in 47 (25.4%) patients. In 82 patients (44.3%), surgery was performed within the 3-year follow-up (Fig 1). The proportion of patients with improvement was not significantly different between the groups (surgical treatment: 51/82, 62.2%; nonsurgical treatment: 57/103, 55.5%; P = 0.28).

The univariable analysis revealed that the duration of illness, types of symptoms, and the presence of each of degenerative spondylolisthesis/scoliosis, hypertension, and depression were significant explanatory variables (P < 0.10) (Table 2). The multivariable analysis with these explanatory factors showed that the absence of cauda equina symptoms (only radicular symptoms) had an odds ratio (OR) of 3.31 (95% confidence interval [CI]: 1.50–7.31); absence of degenerative spondylolisthesis/scoliosis had an OR of 2.53 (95% CI: 1.13–5.65); a <1-year duration of illness had an OR of 3.81 (95% CI: 1.46–9.98); and hypertension had an OR of 2.09 (95% CI: 0.92–4.78) (Table 3).

**Table 2:** Univariable logistic regression analyses for 3-year subjective improvement in lumbar spinal stenosis symptoms through nonsurgical treatment.

Baseline factors	n	Odds ratio(95% CI)	P-value
Age (years)			
<65	40	1.69 (073–3.95)	0.22
65–74	80	1.21 (0.58–2.51)	0.61
≥75	65	1.00	
BMI (kg/m^2^)			
<25	129	088 (0.45–1.73)	0.71
≥25	56	1.00	
Gender			
Female	77	1.19 (0.63–2.25)	0.85
Male	108	1.00	
Educational background (at least a high school graduate)			
Yes	134	1.00	
No	51	0.95 (0.47–1.91)	0.88
Cauda equina symptoms			
Yes	78	1.00	
No	107	4.42 (2.10–9.30)	< 0.001
Degenerative spondylolisthesis/degenerative scoliosis			
Yes	86	1.00	
No	99	2.11 (1.10–4.03)	0.03
Duration of illness (months)			
<12	47	3.68 (1.54–8.81)	0.003
12–59	79	1.72 (0.76–3.89)	0.2
≥60	59	1.00	
Hypertension			
Yes	65	1.00	
No	120	1.96 (0.97–3.95)	0.059
GDS score (tertiles)			
0–2	73	2.07 (0.88–4.14)	0.09
3–6	64	1.73 (0.88–4.83)	0.22
≥7	48	1.00	

BMI, body mass index; GDS, Geriatric Depression Scale; CI, confidence interval

**Table 3:** Multivariable logistic regression analyses for 3-year subjective improvement in lumbar spinal stenosis symptoms through nonsurgical treatment.

Baseline factors	Odds ratio (95% CI)	P-value
Cauda equina symptoms		
Yes	1.00	
No	3.31 (1.50–7.31)	0.003
Degenerative spondylolisthesis/degenerative scoliosis		
Yes	1.00	
No	2.53 (1.13–5.65)	0.024
Duration of illness (months)		
<12	3.81 (1.46–9.98)	0.007
12–59	1.87 (0.77–4.54)	0.17
≥60	1.00	
Hypertension		
Yes	1.00	
No	2.09 (0.92–4.78)	0.08
GDS score (tertiles)		
0–2	2.05 (0.80–5.25)	0.14
3–6	1.80 (0.70–4.68)	0.23
≥7	1.00	

GDS, Geriatric Depression Scale; CI, confidence interval

## Discussion

In patients with LSS from multiple medical centres and varying levels of disease severity and treatments, nonsurgical treatment resulted in subjective improvement of the symptoms at 3 years after enrolment in 30% of the patients; however, in 70% of the patients, the symptoms remained unchanged, worsened, or were treated surgically. Multivariable analysis showed that the factors associated with the improvement of subjective symptoms at 3 years after treatment were the presence of only radicular symptoms, the absence of degenerative spondylolisthesis and scoliosis, and an illness duration of <1 year.

The present study was conducted using a large-scale cohort of LSS patients from multiple medical centres, regardless of the disease severity, resulting in more representative data than previous studies. However, the present study did not include patients with very mild symptoms, who tend not to present at hospitals. Therefore, the prognosis may be slightly different from that in patients with more severe LSS. In addition, the degree of improvement of subjective symptoms was used as the measure of improvement; therefore, there may be differences in the actual improvement. However, the LSS severity is often defined on the basis of the intensity of lower extremity pain, and, because there are no well-defined classifications or criteria, the degree of subjective improvement may be closest to the actual degree of improvement. In a study that compared surgically treated to conservatively treated patients and conducted follow-ups with 19 patients for an average of 31 months, [13] symptoms improved in 30% and remained unchanged in 60% of the conservatively treated patients who did not undergo any procedure. Despite the study’s limitations, including its retrospective nature, unknown inclusion criteria for the conservatively treated patients, and small sample size, the rate of improvement was comparable to that of our cohort. Similarly, in a 5-year follow-up with 120 patients in whom conservative treatment was initially effective, an improvement was found in 43% of patients, the symptoms remained unchanged in 17%, and symptoms worsened in 40% at the final follow-up; however, the patients may have had relatively mild initial symptoms.[6] Moreover, in a prospective, randomised comparative study of surgical treatment for LSS, observations at 10 years after treatment in the 18 patients that received conservative treatment (control group) revealed mild pain in 2 patients (11%), moderate/severe pain in 6 patients (33%), and surgical treatment in 9 patients.[14] At the 2-year follow-up of a randomised cohort study with patients without spinal instability who were identified as surgical candidates and randomised to either surgical or conservative treatment, 43% of the patients with conservative treatment had to be re-assigned to the surgery group, while 28.7% reported an improvement in their symptoms.[15] However, because the patients with improved symptoms did not include those who were converted to the surgery group, it is possible that the percentage would be lower than those in the present study if the percentage was calculated in the same manner. Furthermore, the differences in results in these latter two studies, when compared with the present study, may be explained by the fact that the patients were indicated for surgery and may have had more severe conditions. However, in our study, if long-term follow-up was conducted, the percentage of patients with a favourable prognosis would likely decrease.

There are few reported studies regarding the predictive factors for the subjective improvement of LSS. However, Miyamoto et al. reported that the outcomes were favourable in patients with radicular-type symptoms and in those who showed good improvement after the initial treatment, while outcomes were poor in patients with degenerative scoliosis.[6] Based on our experience, the prognosis of patients with radicular type LSS has been favourable; however, the underlying mechanism is not yet known. In the present study, the percentage of patients with cauda equina deficits whose treatment was converted to surgery was 3 times higher than that of patients with only radicular type LSS, which may support our experience. Degenerative scoliosis/spondylolisthesis was also predictive of poor prognosis in the present study; conservative treatment is reportedly less effective against degenerative scoliosis, [16] including at a 2-year follow-up.[17] It is possible that patients who repeatedly develop radiculopathy symptoms because of a susceptibility to physical compression have a poorer prognosis, and their treatment is likely to be converted to surgery. In addition, long illness duration has been associated with poor surgical outcomes in LSS; [18] likewise, our findings showed that, in the natural course of LSS, illness duration ≥1 year was also a factor for poor prognosis. A long illness duration likely leads to chronic nerve compression, which may cause oedema or Wallerian degeneration of the affected nerves.[19] Although hypertension was not a significant prognostic factor, it tended to be associated with a poor prognosis. Hypertension is more common in patients with LSS than in controls; [20,21] it causes arteriosclerosis and promotes degenerative changes in the spine and intervertebral discs.[22] Because it can also cause chronic obstructive arteriosclerosis, it may aggravate the prognosis; therefore, further studies are needed to determine if hypertension is related with prognosis in LSS.

This study has several limitations. First, because the follow-up rate was 67%, the presence of non-response bias is possible. Second, we intended to exclude lumbar disc herniation with the use of the straight leg raise test. However, the test was often negative in the elderly, even though they had undergone surgery for lumbar disc herniation. Furthermore, disc herniation is often prevalent in degenerative spine and is a concomitant cause of stenosis.[23] Thus, it was difficult to determine whether the cause of lumbar radiculopathy was lumbar disk herniation or LSS in our population, and it is possible that the influence of disk herniation was underestimated. Third, this study collected data at only a single time point, at 3 years from the date of enrolment. Therefore, the results failed to capture the time course of the disease, the rate of improvement, or requirement for surgical treatment. Additionally, we did not control for the nature, intensity, or duration of surgical or nonsurgical management.

The present study, with a wide range of patients with LSS, provided important findings that have not been reported previously and will aid decision-making regarding LSS treatment. In patients with radicular-type symptoms without degenerative scoliosis or spondylolisthesis and an illness duration of <1 year, the prognosis is likely to be favourable; however, in patients with cauda equina symptoms, degenerative scoliosis or spondylolisthesis, and a long disease duration, surgery may need to be proactively considered.

Future long-term follow-up of this cohort should be conducted, potentially with a questionnaire that more accurately measures disease severity and degree of satisfaction, such as the Zurich Claudication Questionnaire developed by Stucki et al., which is currently being used worldwide.[24] Determining the long-term prognosis of LSS may be useful for developing treatment guidelines.

## Conclusion

In 30% of 274 patients with LSS, conservative treatment led to a subjective improvement in the symptoms at the 3-year follow-up; however, in 70% of the patients, the symptoms remained unchanged, worsened, or required surgical treatment. The predictive factors for improved subjective symptoms were the presence of only radicular symptoms, the absence of degenerative spondylolisthesis and scoliosis, and an illness duration of <1 year.

## Supporting Information

S1 File Supporting information. Dataset of this study.
(XLSX)
